# Cochlear implantation impairs intracochlear microcirculation and counteracts iNOS induction in guinea pigs

**DOI:** 10.3389/fncel.2023.1189980

**Published:** 2023-06-28

**Authors:** Benjamin Philipp Ernst, Ulf-Rüdiger Heinrich, Mathias Fries, Regina Meuser, Tobias Rader, Jonas Eckrich, Roland H. Stauber, Sebastian Strieth

**Affiliations:** ^1^Department of Otorhinolaryngology, University Medical Center Bonn (UKB), Bonn, Germany; ^2^Department of Otorhinolaryngology, University Medical Center of the Johannes Gutenberg-University Mainz, Mainz, Germany; ^3^Institute for Medical Biometry, Epidemiology and Informatics (IMBEI), University Medical Center of the Johannes Gutenberg-University Mainz, Mainz, Germany; ^4^Division of Audiology, Department of Otorhinolaryngology, University Hospital, Ludwig-Maximilians-Universität München (LMU), Munich, Germany

**Keywords:** nitric oxide, cochlea implantation, hearing preservation, cochlear microcirculation, microvascular permeability, iNOS

## Abstract

**Introduction:**

Preservation of residual hearing remains a great challenge during cochlear implantation. Cochlear implant (CI) electrode array insertion induces changes in the microvasculature as well as nitric oxide (NO)-dependent vessel dysfunction which have been identified as possible mediators of residual hearing loss after cochlear implantation.

**Methods:**

A total of 24 guinea pigs were randomized to receive either a CI (*n* = 12) or a sham procedure (sham) by performing a cochleostomy without electrode array insertion (*n* = 12). The hearing threshold was determined using frequency-specific compound action potentials. To gain visual access to the stria vascularis, a microscopic window was created in the osseous cochlear lateral wall. Cochlear blood flow (CBF) and cochlear microvascular permeability (CMP) were evaluated immediately after treatment, as well as after 1 and 2 h, respectively. Finally, cochleae were resected for subsequent immunohistochemical analysis of the iNOS expression.

**Results:**

The sham control group showed no change in mean CBF after 1 h (104.2 ± 0.7%) and 2 h (100.8 ± 3.6%) compared to baseline. In contrast, cochlear implantation resulted in a significant continuous decrease in CBF after 1 h (78.8 ± 8.1%, *p* < 0.001) and 2 h (60.6 ± 11.3%, *p* < 0.001). Additionally, the CI group exhibited a significantly increased CMP (+44.9% compared to baseline, *p* < 0.0001) and a significant increase in median hearing threshold (20.4 vs. 2.5 dB SPL, *p* = 0.0009) compared to sham after 2 h. Intriguingly, the CI group showed significantly lower iNOS-expression levels in the organ of Corti (329.5 vs. 54.33 AU, *p* = 0.0003), stria vascularis (596.7 vs. 48.51 AU, *p* < 0.0001), interdental cells (564.0 vs. 109.1 AU, *p* = 0.0003) and limbus fibrocytes (119.4 vs. 18.69 AU, *p* = 0.0286).

**Conclusion:**

Mechanical and NO-dependent microvascular dysfunction seem to play a pivotal role in residual hearing loss after CI electrode array insertion. This may be facilitated by the implantation associated decrease in iNOS expression. Therefore, stabilization of cochlear microcirculation could be a therapeutic strategy to preserve residual hearing.

## 1. Introduction

Cochlear implantation is a well standardized surgical procedure to rehabilitate hearing function in cases of profound hearing loss. The functional replacement of the organ of Corti with a cochlear implant (CI) not only improves the hearing ability itself but reduces social isolation among these patients significantly (Singh et al., 2015; Davis et al., 2016). Particularly in patients undergoing cochlear implantation with functional residual hearing, the preservation of it is of utmost importance not only for electric-acoustic stimulation but also for optimal outcomes in patients with relevant hearing remnants (Ludwig et al., 2021; Tarabichi et al., 2021). Residual hearing preservation in cochlear implantation relies on two principles. Firstly, the surgeon has to adhere to the concept of soft surgery. This includes a round window approach with an atraumatic electrode insertion as well as the perioperative administration of systemically or intratympanically applied corticosteroids (Skarzynski et al., 2011; Kuthubutheen et al., 2015). Secondly, the individual CI electrode array length and design has to be chosen in accordance with the individual patients’ cochlear anatomy and pattern of hearing loss. In case of profound middle to high frequency hearing loss, the electrode array is only inserted into the basal turn of the cochlea. This concept, which is crucial for the electric acoustic stimulation (von Ilberg et al., 1999), protects the medial and apical structures that represent tonotopic areas of residual hearing from direct damage. In some patients, however, residual hearing is either lost immediately or residual hearing decreases several months after the implantation (Balkany et al., 2006; Gstoettner et al., 2006).

Numerous factors are involved in the loss of residual hearing. The electrode array insertion has been shown to cause direct structural damages, including mutilation of the basilar membrane, stria vascularis and the cochlear endosteum. Additionally, fractures of the osseous spiral lamina as well as disruptions of cochlear fluids have been described (Li et al., 2007; Somdas et al., 2007). Moreover, the intracochlear electrode array can trigger direct or indirect inflammatory responses (Roland and Wright, 2006). These may result in hair cell apoptosis due to oxidative stress (Dinh et al., 2011) as well as in the formation of fibrous tissue around the electrode (Foggia et al., 2019) which causes elevation of electrode impedances (Vannini et al., 2015) and subsequent hearing loss (Bas et al., 2016).

In addition, insertion of electrode arrays results in alterations in inner ear fluid pressures which is roughly comparable to high intensity sound exposure regarding its pathophysiological effect (Pau et al., 2007; Todt et al., 2014; Greene et al., 2016). Finally, alterations of the cochlear microcirculation were associated with numerous cochlear pathologies including sudden sensorineural hearing loss (Weiss et al., 2017), noise induced hearing loss (Arpornchayanon et al., 2011) as well as endolymphatic hydrops, which is the accepted pathophysiological disorder underlying Ménière’s disease (Bertlich et al., 2014). The cochlea is principally supplied from the labyrinthine artery, which is a branch of the anterior inferior cerebellar artery (AICA) (Wangemann, 2002; Nakashima et al., 2003). It has been shown that an occlusion of the AICA subsequently results in a reduction of cochlear blood flow (CBF) up to 60% (Ren et al., 1993; Brown and Nuttall, 1994) which may contribute to fibrosis and ossification of the cochlea (Foggia et al., 2019). These findings underline the relevance of blood flow changes in the lateral wall in association with hearing loss after surgical trauma, rather than hair cell loss (Hirose and Liberman, 2003).

The main blood supply to the cochlea is provided by the terminating spiral modiolar artery. This artery has radial branches to the lateral cochlear wall which harbors the two major capillary systems in the spiral ligament and stria vascularis. Four distinct networks are formed by the two capillary systems which are arranged in parallel in the cochlear lateral wall (Nakashima et al., 2003; Shi, 2011). Cochlear blood supply can be regulated at different levels. A strong autoregulation of cochlear microcirculation was detected with a rapid recovery of CBF after occlusion of the anterior inferior cerebellar artery (Nakashima, 1999; Nakashima et al., 2003). In addition, different cell types were identified to be crucial in local blood flow regulation such as smooth muscle cells, pericytes, fibrocytes and local metabolites (Shi, 2011).

Increased noise exposure during the process of electrode array insertion due to fluid pressure elevation induces the formation of intracochlear radicals such as nitric oxide (NO) by the increase of the inducible NO synthase isoform (iNOS) (Shi et al., 2003; Chen et al., 2008; Heinrich and Helling, 2012). NO has been shown to be a key mediator to restore the local microcirculation by various NO-synthases including iNOS. Otherwise, iNOS expression is known to be upregulated following various cytotoxic traumas resulting in a pronounced increase in local NO production (Shah et al., 2020; Yang et al., 2020).

Therefore, this study was designed to investigate the effects of CI electrode array insertion on microvascular parameters and the extent of iNOS induction in the guinea pig cochlea in the various cell types of the inner ear.

## 2. Materials and methods

### 2.1. Animals

All animal experiments were conducted in compliance with the arrive guidelines as well as with the German Prevention of Cruelty to Animals Act and were approved by the responsible supervising authorities in Koblenz, Rhineland-Palatinate (Landesuntersuchungsamt Rheinland-Pfalz; license no. 23 177-07/G07-1-010).

A total of 24 healthy female albino guinea pigs (Dunkin Hartley, Charles River, Sulzfeld, Germany) at age 8–10 weeks, weighing 250–300 g with good Preyer’s reflexes and no evidence of middle ear disease were used in this study. Animals were kept on a 12 h:12 h light:dark cycle in the university’s animal facility for acclimation 1 week prior to inclusion.

### 2.2. Surgical intervention

The guinea pigs were anesthetized with intraperitoneal injections of esketamine hydrochloride (Ketanest, Pfizer, Karlsruhe, Germany; 85 mg/kg body weight) and xylazine hydrochloride (Rompun, Bayer, Leverkusen, Germany; 8,5 mg/kg body weight). Total anesthesia was maintained by repeated intraperitoneal injections of esketamine hydrochloride (42.5 mg/kg body weight) and xylazine hydrochloride (4.25 mg/kg body weight). Additionally, articaine hydrochloride (Ultracain 1%, Sanofi, Paris, France, 0.5 ml) was injected into the area of surgical preparation to achieve additional local anesthesia in order to reduce the amounts of systemic anesthetics. Animals were randomized to receive either a CI insertion (*n* = 12) or a sham procedure consisting of a cochleostomy without insertion (*n* = 12). Surgical preparation lasted for about 45 min. After induction of a sufficient level of anesthesia, animals were placed on a temperature-regulated warming pad. A central venous catheter (CVC) was placed in the left jugular vein for fluid replacement (NaCl 0.9%, 80 μl/kg bodyweight/min) and injection of fluorescein markers. Animals were fixed using a guinea pig-specific non-traumatic head holder (SH-15, Tritech Research, Los Angeles, CA, USA). Surgical exposure of the cochlea, cochleostomy, electrode array insertion and creation of a bony window to the lateral cochlear wall for fluorescence microscopy were performed as previously described (Arpornchayanon et al., 2013; Honeder et al., 2018).

In a first step, the auricle was resected, the temporal bone and the auditory bulla were exposed. The lateral aspect of the auditory bulla was removed to expose the cochlea. The facial nerve was identified and conserved. Vessels of the cochlear periost were gently removed by hydro-dissection. An insulated 0.125 mm gold probe (Goodfellow, Friedberg, Germany) serving as the anode was mounted to the round window membrane and fixed to the temporal bone for detection of evoked auditory responses. Additional silver probes (0.25 mm, Goodfellow, Friedberg, Germany) were introduced subcutaneously at the vertex (cathode) as well as at the lumbar region (grounding). Before cochleostomy and electrode insertion, baseline hearing thresholds were measured by frequency-specific compound action potentials (CAP). Cochleostomy in the scala tympani of the basal cochlear turn was performed using a 0.6 mm diamond burr at the lowest possible rotational frequency. Depending on the randomization, a solitary cochleostomy (sham) or cochleostomy with subsequent customized CI electrode array insertion (Med-El, Innsbruck, Austria, 4 contact experimental guinea pig electrodes, insertion depth of 4 mm) were carried out, respectively.

**FIGURE 1:**
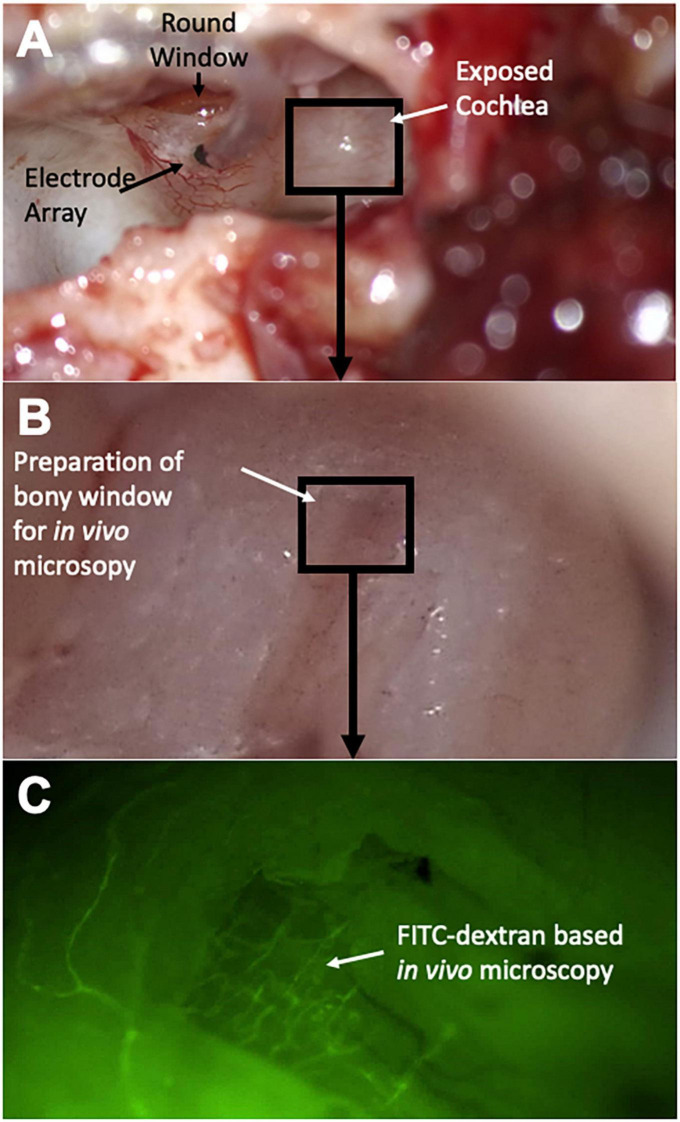
Surgical steps and experimental setup. After resection of the auricle, the lateral aspect of the auditory bulla is removed to expose the cochlea **(A)**. The round window niche is identified and a cochleostomy is introduced into the basal turn of the cochlea for subsequent electrode array insertion. In a next step, a bony window is introduced in the second turn of the cochlea **(B)** for subsequent *in vivo* microscopy **(C)**; FITC dextran infusion highlights microvessels of the cochlear lateral wall.

In a next step, a 0.5 × 0.5 mm bony window flap was elevated in order to gain visual access to the stria vascularis for subsequent fluorescence *in vivo* microscopy (see Figure 1).

**FIGURE 2:**
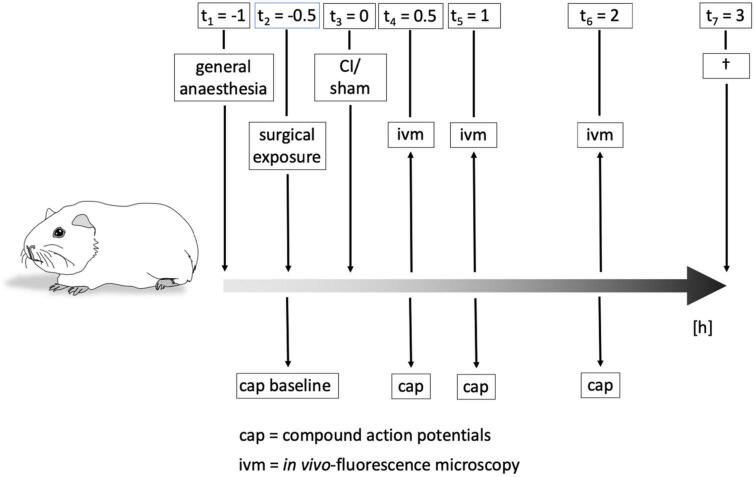
Time course of experiments. Animals were put under general anesthesia about 1 h before cochlear implant (CI) or sham procedure, respectively. Surgical exposure of the cochlea and evaluation of baseline hearing threshold by compound action potential (CAP) were performed before the intervention. CAPs and *in vivo*-fluorescence microscopy (IVM) to determine cochlear microcirculation were carried out immediately after CI/sham as well as after 1 h and 2 h. After all measurements, animals were killed and the cochleae removed for subsequent immunohistochemical examinations.

Animals were kept under total anesthesia after cochleostomy and electrode insertion for another 2 h for repetitive measurements. See Figure 2 for more details concerning the time schedule of the experiments.

### 2.3. Evaluation of auditory function

Hearing thresholds were evaluated before intervention by recording frequency-compound action potentials as previously described (Muller et al., 2017; Honeder et al., 2019). Auditory testing was carried out in a soundproof chamber. A custom-made microphone as well as a custom-made speaker were placed at equal distances of about 2.5 cm from the external auditory canal to be tested. The opposite external auditory canal was sealed using a soft ear wax plug (Ohropax, Werheim, Germany). A computer-based setup incorporating a multifunction I/O card (National Instruments Corp., Austin, TX, USA) and AudiologyLab 3.8 (Otoconsult, Frankfurt am Main, Germany) were used for measurements of frequency specific CAP thresholds. Three dB- increments were chosen between 1 and 32 kHz before and immediately after the intervention as well as after 1 and 2 h. Threshold shifts were determined as the difference in pre- and post-interventional frequency-specific CAP thresholds (Δ hearing threshold_*sham*_; Δ hearing threshold_*CI*_).

### 2.4. *In vivo*-microscopy for analysis of cochlear microcirculation

*In vivo*-fluorescence microscopy (IVM) was utilized to analyze CBF and cochlear microvascular permeability (CMP). To determine CBF, 0.1 mL fluorescein isothiocyanate (FITC)-labeled dextran (Sigma, Deisenhofen, Germany; average molecular weight of 500,000; 5% solution in 0.9% NaCl) was applied via the CVC in order to increase the contrast between blood cells and blood plasma. For analysis of CMP, 0.1 mL of Tetramethylrhodamine isothiocyanate (TRITC)-labeled dextran (Sigma, Deisenhofen, Germany; average molecular weight of 60,000–70,000; 5% solution in 0.9% NaCl) was injected via the CVC.

For IVM measurements, animals were put on the counter lateral side with heads fixed to the head holder to ensure immobility. To allow microscopical analysis, the ear was aligned upward toward the microscope (Olympus BXFM, Olympus Deutschland GmbH, Hamburg, Germany). Microscopical investigation was performed using a long-distance objective (Olympus Deutschland GmbH, Hamburg, Germany) with an overall magnification of 156×. After verification of adequate exposure of the microvessels within the stria vascularis, video sequences were digitally captured by means of a green filter (Excitation: 470 nm; Emission: 525 nm) for FITC-labeled dextran-enhanced IVM or an orange filter (Excitation: 545 nm; Emission: 605 nm) for TRITC-labeled dextran-enhanced IVM, respectively. Video sequences underwent processing using the built-in Cell Sens Dimension software (Olympus Deutschland GmbH, Hamburg, Germany) and were stored as uncompressed avi-files for subsequent off-line analysis.

Off-line analysis of CBF was performed utilizing an off-line computer-aided videoframe analysis software (Cap Image, Dr. Zeintl Ingenieurbüro, Dreieich, Germany) (Arpornchayanon et al., 2013; Eckrich et al., 2021a). For each cochlear window, vessel diameter (d) and blood flow velocity (V_*RBC*_) were evaluated for 3–4 independent vessels and the CBF was calculated as previously described by Baker and Wayland (1974):


$$\mathrm{CBF}=\left(V_{\mathrm{RBC}}/1.6\right)\times {\left(d/2\right)}^{2}\times \pi$$

Off-line analysis of CMP was carried out using the Cell Sens Dimension software. Regions of interest were defined including the intra- and extravasal space. Analysis of fluorescence intensity for CMP measurements was carried before application of TRITC-labeled dextran (baseline) as well as every 30 s for 20 min afterwards with regard to background fluorescence, respectively. To allow the quantification of CMP, the mean fluorescence intensity [arbitrary units (AU)] was measured for various regions of Interest (ROI) within the vascular network as well as for the surrounding tissue. Thus, relative changes of intra- and extravasal fluorescence intensities were monitored over time and quantified (d/2)^2^.

### 2.5. Measurement of mean arterial pressure, pulse rate, oxygen saturation levels, and body temperature

In order to control stable and comparable macrocirculatory prerequisites, mean arterial blood pressure (MAP), heart rate (HR) and blood oxygen saturation level were monitored in both groups continuously. In accordance with the literature, minimum values for inclusion were defined as MAP of 40 mmHg, HR of 190 beats per minute (bpm) and blood oxygen saturation level of 97% (Brown et al., 1989; Scheibe et al., 1989; Canis et al., 2010; Arpornchayanon et al., 2011; Schmitz et al., 2016). Body temperature was maintained at 38°C using a temperature-regulated heating pad. Minimum body temperature for inclusion was 37°C. Animals with pathologic parameters were excluded from the study. Before each measurement, values were documented to rule out potential effects on microcirculation.

Mean arterial pressure was measured non-invasively in the right posterior limb (Murom chi Non-Invasive Blood Pressure Monitor, Steeling Co., Wood Dale, IL, USA). Pulse rate and oxygen saturation level were measured using a veterinary pulse oximeter (PM-60Vet, Mindray, Shenzhen, China). Body temperature was assessed using a rectal thermometer (FT09, Beurer GmbH, Ulm, Germany).

### 2.6. Extraction and fixation of cochleae

After completion of all measurements, total anesthesia was deepened and animals were killed by intravenous injection of an overdose of anesthetics.

The bullae were removed from the bone and transferred into a solution that consisted of 0.2% picric acid, 4% paraformaldehyde and 0.1% glutardialdehyde. iNOS distribution was verified by polyclonal rabbit IgG anti-iNOS antibodies (BML-SA200, Enzo Life Sciences, Farmingdale, NY, USA, diluted 1:2000). Sections from different experimental ears were collected on different slices. During each staining procedure, control ears were stained side by side. Comparing different immunostaining procedures and different slides of the same ear, comparable values were obtained (Heinrich et al., 2010).

### 2.7. Quantification of iNOS immunostaining intensity

Quantification of immunostaining intensity in various regions of the cochlea was carried out as previously described (Heinrich et al., 2010). These regions included interdental cells, fibrocytes of the limbus, nerve fibers, organ of Corti, stria vascularis and the spiral ligament. Briefly, images were taken from semi-thin sections using a TE-DIH-F camera (Nikon Europe BV, Amsterdam, Netherlands) connected to an Eclipse TE2000-U microscope (Nikon Europe BV, Amsterdam, Netherlands) equipped with a halogen light source using a 40× objective. Images were captured digitally at a total magnification of 400× and processed using Photoshop 7 (Adobe Systems, San Jose, CA, USA). Areas of interest were marked and staining intensities were quantified.

### 2.8. Statistical analysis

Data of CBF and CMP are presented as the mean ± standard deviation (SD) while data of auditory testing and immunohistochemistry are presented as the median with interquartile range. Kolmogorov-Smirnov test was used to determine normal distribution. Unpaired *t*-test was used to CBF and CMP. Wilcoxon rank-sum test was used to compare results of auditory testing and immunohistochemistry. Analysis was stratified by cell type. Each animal contributed three observations, one for each turn. The endpoint was defined as the iNOS immunostaining intensity. Statistical analysis was performed using Prism 9 (GraphPad Software, La Jolla, California, USA).

## 3. Results

### 3.1. Cardiovascular parameters

Cardiovascular parameters remained stable throughout the experiments. Mean MAP was 47.9 ± 5.7 mmHg for the sham group and 46.8 ± 4.3 mmHg for the CI group (*p* = 0.5126). Mean heart rate was 205.6 ± 13.0 bpm for the sham group and 204.0 ± 11.9 bpm for the CI group (*p* = 0.6885). Mean blood oxygen saturation levels were 98.5 ± 1.0% for the sham group and 98.5 ± 1.1% for the CI group (*p* = 0.9012). Mean rectal body temperature was 38.1 ± 0.4°C for the sham group and 38.0 ± 0.4°C for the CI group (*p* = 0.8834).

### 3.2. Hearing loss

**FIGURE 3:**
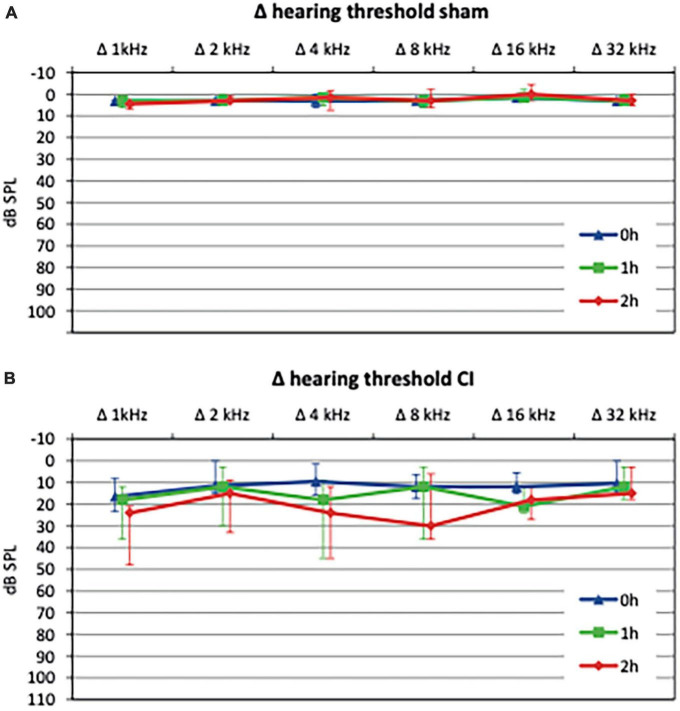
Hearing thresholds shifts relative to baseline values after sham procedure or cochlear implant (CI) electrode array insertion, respectively. Sham procedure, consisting of a cochleostomy alone, did not cause a significant threshold shift up to 2 h postoperatively **(A)**. In contrast, CI electrode array insertions lead to a significant hearing loss immediately after implantation (0 h) which progressed after 1 h and 2 h **(B)**.

Evaluation of baseline hearing thresholds using CAPs was carried out before the intervention (baseline value) as well as after 1 h and 2 h and compared to baseline values (Δ hearing threshold, see Figure 3). After sham procedure, no significant change in mean hearing threshold compared to baseline was observed (40.5 vs. 44.75 dB SPL, *p* = 0.59, see Figure 3A). In contrast, electrode array insertion resulted in a significant threshold shift (Δ hearing threshold) compared to sham control postoperatively (4.1 vs. 12.6 dB SPL, *p* = 0.017) with a progression after 1 h (3.2 vs. 16.6 dB SPL, *p* = 0.0004) and 2 h (2.5 vs. 20.4 dB SPL, *p* < 0.0001, see Figure 3B).

### 3.3. Cochlear blood flow

Evaluation of CBF was carried out by FITC-labeled dextran (500 kDa)-enhanced IVM.

**FIGURE 4:**
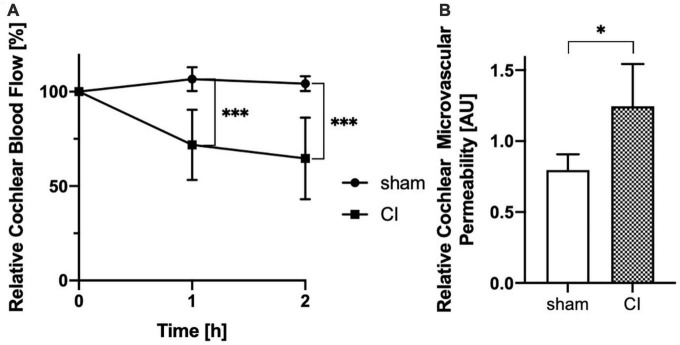
Evaluation of cochlear blood flow (CBF) shows a progressive decrease after cochlear implant (CI) electrode array insertion after 1 h and 2 h comparing with sham controls **(A)**. Analysis of cochlear microvascular permeability (CMP) reveals a significant increase in permeability after cochlear implant (CI) electrode array insertion when compared to sham control **(B)**. *p < 0.05, ^***^p < 0.001.

While mean CBF remained very stable following sham procedure, cochlear implant electrode array insertion caused a significant and progressive decrease after 1 h (106.6 vs. 71.83%, *p* = 0.0009) and 2 h (104.2 vs. 64.61%, *p* = 0.0009, see Figure 4A) compared to baseline values.

### 3.4. Cochlear microvascular permeability

Evaluation of CMP was carried out by TRITC-labeled dextran (60–70 kDa)-enhanced IVM. Continuous monitoring ensured stable and comparable arterial pressure, pulse rate and oxygen saturation levels.

Cochlear microvascular permeability was evaluated for by repetitive determination of the intra- and extravasal fluorescence intensities before and up to 20 min after bolus injection of 60–70 kDa TRITC-dextran. Following bolus injection, intravasal fluorescence analysis revealed a spike which faded gradually over time. Additionally, extravasal fluorescence intensity gradually increased. Comparative analysis of fluorescence intensities of the intra- and extravasal space revealed a significant increase in extravasal fluorescence following CI electrode array insertion compared to sham (1.247 vs. 0.8 AU, *p* = 0.0296, see Figure 4B).

### 3.5. iNOS distribution

#### 3.5.1. Localization of iNOS immunoreaction

**FIGURE 5:**
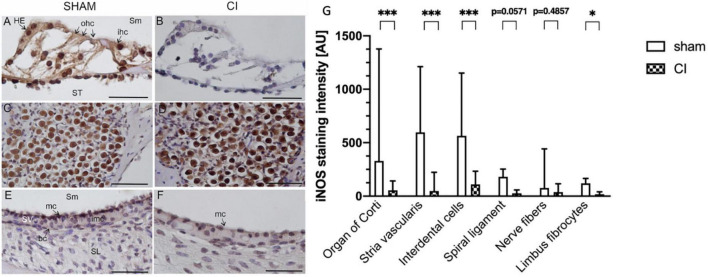
Immunohistochemical anti-iNOS staining within the second turn of the cochlea after sham **(A,C,E)** and CI **(B,D,F)**. Anti-iNOS immunoreaction was identified in various cell types in the organ of Corti after sham **(A)**, whereas no reaction was identified after CI **(B)**. In spiral ganglion cells, a pronounced staining intensity was detected after sham **(C)** as well as and CI **(D)**. In the marginal cells (mc) of the stria vascularis, a clear anti-iNOS staining was detectable after sham **(E)**, which was nearly absent after CI **(F)**. Quantification of anti-iNOS staining intensities in six distinct cell types after sham CI electrode array insertion **(G)**. Compared to sham, CI electrode array insertion resulted in significantly reduced staining intensities in the organ of Corti, stria vascularis, interdental cells, and limbus fibrocytes. All other cell types showed similar tendencies without reaching statistical significance. bc, basal cells; ohc, outer hair cells; HE, Hensen cells; imc, intermediate cells; ihc, inner hair cells; mc, marginal cells; SL, spiral ligament; Sm, scala media; SV, stria vascularis; ST, scala tympani; scale bar = 50 nm. *p < 0.05, ^***^p < 0.001.

In immunohistochemical slides stained with an anti-iNOS antibody, iNOS expression was observed after sham and CI in all cochleae. In the organ of Corti, an increase in iNOS expression was apparent in various sensory and non-sensory cell types (see Figures 5A, B). An intense immunoreaction was primarily detected in inner hair cells and Hensen cells, but could also be localized in outer hair cells (Figure 5A). Moreover, iNOS expression was observed in spiral ganglion cells after both sham and CI treatment (Figures 5C, D). In the lateral wall, differences in staining intensities were mainly observed in marginal cells facing the scala media (Figures 5E, F). Here, increased anti-iNOS staining intensities were detectable after sham (Figure 5E), whilst only very few cells showed a detectable anti-iNOS staining after CI (Figure 5F).

#### 3.5.2. Quantification of iNOS immunoreaction

In order to gain more insights into the differential cellular expression patterns of iNOS within the cochlea, the staining intensity was quantified in 6 distinct cochlear regions. These regions included interdental cells, fibrocytes of the limbus, nerve fibers, organ of Corti, stria vascularis and the spiral ligament. The quantification of iNOS immunoreaction revealed significantly reduced iNOS immunoreactions in the organ of Corti (329.5 vs. 54.33 AU, *p* = 0.0003), stria vascularis (596.7 vs. 48.51 AU, *p* < 0.0001), interdental cells (564.0 vs. 109.1 AU, *p* = 0.0003) and limbus fibrocytes (119.4 vs. 18.69 AU, *p* = 0.0286) after electrode array insertion (see Figure 5G). The other analyzed cochlear regions showed a tendency towards a decreased iNOS immunoreactivity without reaching statistical significance.

## 4. Discussion

Loss of residual hearing after CI is still a clinically relevant problem in modern cochlear implant surgery, especially in patients who are suitable for electric acoustic stimulation (Ludwig et al., 2021; Tarabichi et al., 2021). In concordance with these established clinical findings, the experimental data of the present study showed significant and progressive hearing loss 1 h and 2 h after CI electrode array insertion compared to sham. Additionally, there was evidence for a severe impairment of intracochlear microcirculation accompanied by inherent cochlear protection mechanisms.

### 4.1. Electrode array insertion associated hearing loss

Hearing loss causes following cochlear implantation can be divided into immediate and delayed types. Immediate hearing loss occurs directly after electrode array insertion and is likely caused by mechanical forces potentially leading to scalar translocation (Shaul et al., 2018; Jwair et al., 2021). Consistent with findings by Jia et al., disturbances in blood flow following CI electrode array insertion were observed (Jia et al., 2013). The insertion can lead to distress of the cochlear microcirculation preventing oxygen and nutrient supply as well induction of inflammatory responses and the alteration of the cochlear potential as highlighted by the present data (Jia et al., 2013). Consequently, alterations within the stria vascularis capillary structure and perfusion have been associated with immediate hearing loss after implantation (Reiss et al., 2015). These experimental findings emphasize the importance of lateral wall blood flow changes, rather than implantation-associated hair cell losses, in immediate type residual hearing loss after CI (Tejani et al., 2021).

Delayed hearing loss following CI has also been attributed to the delayed and progressive effects due to the mechanical trauma of the electrode array insertion (Roland and Wright, 2006) as well as concomitant physiological alterations such as a proinflammatory immune response due to lateral wall impairment induced by the presence of the CI electrode array (Rai et al., 2020). The inflammation may ultimately result in intracochlear fibrosis and sclerosis (Foggia et al., 2019) causing elevation of electrode impedances (Vannini et al., 2015) as well as to progressive hearing loss due to ongoing hair cell loss (Vivero et al., 2008).

### 4.2. Impairment of intracochlear microcirculation following electrode array insertion

The presented data show that immediate hearing loss following cochlear implantation is accompanied by a significantly reduced blood flow in the lateral wall and an increased vascular permeability. In the context of cochlear implantation, the altered blood flow may result from a reduced blood influx via the modiolar artery, a damage or leakage of the blood vessels within the vascular network of the cochlear lateral wall or a disturbed venous drainage due to a downstream occlusion. Many otologic disorders such as noise-induced hearing loss, endolymphatic hydrops and presbycusis have been identified to be related to alterations in CBF (Nakashima et al., 2003). Additionally, impairment of CBF is likely to also play a pivotal role in loss of residual hearing after CI (McClellan et al., 2021).

Due to their position inside the cochlea, lateral wall CI electrode arrays exert direct mechanical pressure to the underlying tissue (Wright and Roland, 2013). The vein of the cochlear aqueduct is the principal draining vein of the cochlea in the guinea pig (Watanabe et al., 1988). More specifically, the venous system in the lateral wall of the scala tympani drains the blood flow from both the stria vascularis and the modiolus (Wright and Roland, 2013; McClellan et al., 2021). These vessels have little or no bony coverage which leaves them largely exposed to the perilymphatic space. Here, they are at risk to be mechanically traumatized by shear forces and to be compressed by the electrode array (Wright and Roland, 2013). Impairment of the venous outflow of the cochlear lateral wall causes venous stasis and consecutive backlog in the capillary system of the stria vascularis (Wright and Roland, 2013).

There is strong evidence for an autoregulation of CBF within the lateral wall (Nakashima, 1999) which may modulate arterial blood inflow in response to changes in postcapillary pressure (Brown and Nuttall, 1994; Nakashima, 1999). Consequently, increases of the venous pressure within the stria vascularis may trigger an unfavorable autoregulatory response which causes a decrease in CBF (see Figure 4A). Additionally, venous stasis likely results in an increase in CMP due to the pressure increase within the capillary bed of the stria vascularis as shown in the presented data. Additionally, CMP may also be increased by direct damages of the CI electrode array insertion to the lateral wall of the cochlea.

As outlined by various publications, CI electrode array insertions cause an elevation of the endocochlear pressure comparable to high intensity noise exposure (Pau et al., 2007; Todt et al., 2014; Greene et al., 2016). Noise exposure has been shown to alter cochlear microcirculation and trigger immunological responses within the cochlea as well as systemically (Arpornchayanon et al., 2011; Shi, 2011; Eckrich et al., 2021b). Previous research by our group showed that red blood cell velocity and consecutively the segmental CBF was reduced significantly after noise exposure (Arpornchayanon et al., 2011). As the cochlea demands a constant supply of oxygen and nutrients, the labyrinthine artery function is closely linked to proper homeostasis especially in noisy conditions (Zhao et al., 2006; Arpornchayanon et al., 2011).

Alterations of microvascular parameters within this study may be caused by a combination of a venous pressure increase within the capillary bed of the stria vascularis in combination with an elevated intracochlear pressure due to the electrode insertion leading to a dysfunction of arterial influx. Both have been associated with the breakdown of the endocochlear potential with a consecutive loss of residual hearing (McClellan et al., 2021).

### 4.3. Inhibition of cochlear iNOS induction following cochlear implantation

Inducible NO synthase isoform expression has been shown to be highly relevant in cochlear inflammatory response (Hess et al., 1999; Watanabe et al., 2000; Shi et al., 2003), vascular trauma (Zhou et al., 2021) and, as a potent regulator of NO production (Heinrich and Helling, 2012), control of blood flow (Brechtelsbauer et al., 1994; Yamane et al., 1997). Hence, spatial and quantitative cochlear iNOS expression was analyzed in this study.

Inducible NO synthase isoform expression was detected throughout all analyzed specimen in which the sham group showed a significantly stronger upregulation, especially in the organ of Corti, stria vascularis, interdental cells and limbus fibrocytes, compared to the CI group.

Various factors including trauma and increase in endocochlear pressure have been shown to modulate the expression if iNOS and consequently the amount of NO production (Shi et al., 2003; Chen et al., 2008; Heinrich and Helling, 2012). In consequence, there is a high relevance with regard to NO-dependent microcirculation. iNOS expression is typically not found in untreated cochlear tissue (Gosepath et al., 1997). However, iNOS expression is upregulated in the cochlea after various stimuli including trauma and ischemia (Shi and Nuttall, 2003; Morizane et al., 2005; Inai et al., 2012). Based on these findings, a strong upregulation in cochlear iNOS expression should be expected following CI electrode array insertion. In this regard, various publications show an elevated iNOS expression following noise exposure, comparable to the burr-dependent cochleostomy, mainly in hair cells, strial marginal cells and in the stria vascularis (Shi et al., 2003; Inai et al., 2012). This leads to marginal cell pathology and dysfunction of cochlear microcirculation which may be evoked by blood vessel wall damage (Shi and Nuttall, 2003).

As the degree of traumatization of combined cochleostomy and CI electrode array insertion was higher in the CI group, iNOS upregulation was anticipated to be greater. However, while all specimen exhibited an upregulation of iNOS, overall expression was significantly higher in the sham group compared to CI. Consistent with previous publications, iNOS upregulation was pronounced in the organ of Corti, the interdental cells as well as the stria vascularis and limbus fibrocytes, whereas only a marginal upregulation was detected after CI. iNOS is well known to synthesize high amounts of NO continuously until the enzyme is degraded (Kolodziejski et al., 2002). Therefore, an impairment in iNOS induction due to the CI electrode array insertion as well as an accelerated degradation of iNOS is possible. Both effects are likely to contribute to the reduced CBF. As models of ischemia in neuronal systems were able to demonstrate a severe dysregulation of proteostasis causing a significant decrease in protein synthesis (Thiebaut et al., 2019), similar effects can be expected for iNOS.

Inducible NO synthase isoform expression levels have been shown to be influenced by a variety of processes. Due to the early onset of reduced iNOS expression levels 2 h following cochlear implantation, iNOS degradation by ubiquitination (Kolodziejski et al., 2002) or alterations of iNOS mRNA stability (Korhonen et al., 2002; Lisi et al., 2011) are not likely.

Instead, fast-acting regulatory systems such as auto-phagocytosis (Wang et al., 2019) as well as NO-dependent auto-inactivation of iNOS (Cinelli et al., 2020) are potential key regulators in this scenario.

Macrophages exert a pivotal role in homeostasis of various processes within the cochlea including response to cochlear injury (Hirose et al., 2005; He et al., 2020). In the unstimulated cochlea, macrophages were identified the spiral ligament, spiral limbus, spiral ganglion region, osseous spiral lamina, basilar membrane, as well as around the blood vessels of the stria vascularis (Hirose et al., 2005; Shi, 2010). In this scenario, macrophages have a dual and potentially antagonizing role, as they are activated by various stimuli to protect the cochlea by significantly increased production of NO (Aktan, 2004; Laskin et al., 2011). In contrary, macrophages are also able to decrease NO levels by iNOS-degradation in the context of autophagy (Wang et al., 2019). Generally, autophagy is an intracellular lysosome-dependent process which aims to degradate cellular components (Klionsky, 2008). This process works selectively via the autophagy receptor proteins (Liu et al., 2014). For the cochlea, basal autophagy has an important role in maintenance of hair cell morphology as well as hearing capacity and sensitivity (Hayashi et al., 2020; Guo et al., 2021). iNOS interacts with the autophagy receptor p62 and is degraded by autophagy in macrophages in order to modulate NO production during inflammation (Wang et al., 2019). Thus, macrophage-dependent autophagy may contribute to the reduced iNOS expression following CI in the present study. An increase in the density of macrophages in the cochlea after cochlear implantation was demonstrated recently (Okayasu et al., 2020) which may be grounds for intracochlear fibrosis and sclerosis in the long run (Jia et al., 2013; Wilk et al., 2016; Simoni et al., 2020).

Furthermore, NO itself may downregulate iNOS activity, representing a process of auto-inactivation (Cinelli et al., 2020). It was shown that the expression of iNOS could be directly influenced by the local amount of NO itself. The NO donors S-nitroso-N-acetyl-D,L-penicillamine (SNAP) and V-PYRRO/NO reduce iNOS promoter activity and consequently the iNOS-expression levels significantly (Chang et al., 2004). A decrease in CBF may trigger NO production by the constitutively expressed endothelial nitric oxide synthase (eNOS) and neuronal nitric oxide synthase (nNOS) isoenzymes. Analog to iNOS, hemodynamic factors and mechanic forces within the vasculature were found to influence the regulation of the constitutively expressed eNOS-isoform, thereby altering the level of NO-concentration (Yamamoto et al., 1998; Kuebler et al., 2003). Hence, the increased NO production by NOS isoforms may contribute to an excess in NO which leads to a fast degradation of iNOS. In both scenarios, overall amounts of intracochlear NO, due to iNOS degradation by autophagy or auto-inactivation, play a central role.

Changes in CBF, iNOS expression and overall NO production are potentially linked to inherent protection mechanisms within the cochlea. The posttranscriptional regulation of iNOS expression by mRNA as well as protein degradation (Walker et al., 1997; Korhonen et al., 2002) are also closely linked to the activity of glucocorticosteroids, which are routinely used in cochlear implantation in order to promote residual hearing preservation (Skarzynska et al., 2018). Additionally, NO homeostasis has been associated with other inherent cellular protection mechanisms in a variety of diseases (Vannini et al., 2015). In various models of upper and lower airway cancer, application of the NO donors SNAP and sodium nitroprusside or induction of iNOS have been shown to modulate cell growth, apoptosis and the expression of survivin (Chao et al., 2004; Fetz et al., 2009). Survivin has been identified as an apoptosis inhibitor and a mitotic regulator which is able to protect cells against caspase-dependent as well as -independent cell death (Altieri, 2008). Modulation of these characteristics make survivin an interesting target regarding otoprotection. The otoprotective role of survivin in the guinea pig model after moderate noise exposure has been demonstrated through its Akt-mediated increase in expression by others (Habtemichael et al., 2010; Knauer et al., 2010). In accordance with the literature, there is a possible crosstalk between iNOS and survivin through the transcription factor signal transducer and activator of transcription 3 (STAT3). STAT3 regulates transcription of pro-survival target genes such as survivin (Gritsko et al., 2006) and also binds directly to the promoter of the iNOS gene and thereby stimulates its expression (Jahani-Asl and Bonni, 2013). Consequently, the survivin pathway can be considered as an indicator of cell stress as well as part of the inherent cochlear protection mechanism following trauma with links to the NO pathway.

## 5. Conclusion

In summary, microvascular dysfunction in the lateral wall of the cochlea due to alterations in NO-dependent signaling pathways as well as direct mechanical trauma seems to play a pivotal role in residual hearing loss after cochlear implantation. Following cochlear implantation, microvascular dysfunction is promoted by the decrease in iNOS expression as shown in the present study and the associated decrease in overall NO production. Therefore, NO-stabilizing perioperative therapies like inhalative application of NO or pharmacological induction of iNOS (Palikhe et al., 2019; Yu et al., 2019) have to be considered as a therapeutic regimen for residual hearing preservation. Additionally, there is a rationale for targeted therapies by exploiting the evident increased CMP which enables a local accumulation of pharmacological agents within the traumatized cochlea. By stabilizing overall NO production and iNOS expression, induction of cellular defense mechanisms, such as the survivin pathway, may be facilitated. This may lead to a decrease in cellular stress and potential cell death, which potentially contributes to residual hearing preservation in cochlear implantation.

## Data availability statement

The raw data supporting the conclusions of this article will be made available by the authors, without undue reservation.

## Ethics statement

The animal study was reviewed and approved by Landesuntersuchungsamt Rheinland-Pfalz, Mainzer Straße 112, 56068 Koblenz, Germany (license no. 23 177-07/G07-1-010).

## Author contributions

BE, RS, and SS contributed to the conception and design of the study. SS performed the funding acquisition. BE and JE carried out the surgical procedures. BE, U-RH, and MF performed the histopathological analysis. BE, RM, and TR performed the statistical analysis. BE, U-RH, MF, RM, TR, JE, RS, and SS wrote sections of the manuscript. All authors contributed to the manuscript revision and read and approved the submitted version.
